# What Do We Know about How Hantaviruses Interact with Their Different Hosts?

**DOI:** 10.3390/v8080223

**Published:** 2016-08-11

**Authors:** Myriam Ermonval, Florence Baychelier, Noël Tordo

**Affiliations:** Unité des Stratégies Antivirales, Département de Virologie, Institut Pasteur, 25 Rue du Docteur Roux, 75015 Paris, France; Florence.Baychelier-tine@sanofi.com (F.B.); ntordo@pasteur.fr (N.T.)

**Keywords:** *Bunyaviridae*, hantavirus, virus-host interaction, rodents, humans, insectivores

## Abstract

Hantaviruses, like other members of the *Bunyaviridae* family, are emerging viruses that are able to cause hemorrhagic fevers. Occasional transmission to humans is due to inhalation of contaminated aerosolized excreta from infected rodents. Hantaviruses are asymptomatic in their rodent or insectivore natural hosts with which they have co-evolved for millions of years. In contrast, hantaviruses cause different pathologies in humans with varying mortality rates, depending on the hantavirus species and its geographic origin. Cases of hemorrhagic fever with renal syndrome (HFRS) have been reported in Europe and Asia, while hantavirus cardiopulmonary syndromes (HCPS) are observed in the Americas. In some cases, diseases caused by Old World hantaviruses exhibit HCPS-like symptoms. Although the etiologic agents of HFRS were identified in the early 1980s, the way hantaviruses interact with their different hosts still remains elusive. What are the entry receptors? How do hantaviruses propagate in the organism and how do they cope with the immune system? This review summarizes recent data documenting interactions established by pathogenic and nonpathogenic hantaviruses with their natural or human hosts that could highlight their different outcomes.

## 1. Introduction

Hantaviruses are tri-segmented, enveloped, RNA viruses of negative polarity, belonging to the family *Bunyaviridae*. In contrast to members of the four other genera, viruses from the Hantavirus genus are not arthropod borne. Their human transmission occurs through inhalation of aerosolized virus particles present in dried excreta of naturally infected rodents, among which, hantaviruses circulate without giving any recognized symptoms. Human pathologies linked to hantaviruses have been observed since the 1950s, with the documentation of Korean Hemorrhagic Fever, but the Hantaan virus (HTNV) prototype was only isolated from the striped field mouse (*Apodemus agrarius*) in 1978 [1,2]. To date, only hantaviruses circulating among rodent reservoirs (Murinae, Arvicolinae, and Sigmodontinae) have been found to be pathogenic to humans.

New hantaviruses are now described in many small insectivorous mammals [3], such as moles and shrews (Soricomorpha), as well as in bats (Chiroptera). A recent study performed in Brazilian bats showed the presence of hantaviruses among different bats species, and not solely in insectivorous bats as previously suggested [4]. These discoveries point to the fact that hantaviruses circulate in host reservoirs present worldwide, extending the risk of possible host co-infection by different hantaviruses, and therefore virus reassortment and host spill-over. Moreover, changes in reservoir ecology due to human impacts on climate and biodiversity are additional factors that make hantaviruses a global public health concern [5,6].

Since their initial discovery, more than 20 different species of hantaviruses that are pathogenic for humans have been described, with new viruses regularly found all over the world. They cause hemorrhagic fever with renal syndrome (HFRS) in the Old World (Europe and Asia) while hantavirus cardiopulmonary syndrome (HCPS) is more specifically associated with human diseases in the New World (Americas). The major HFRS symptoms are acute kidney injury (AKI) and hemorrhagic fevers.

The most frequent hantavirus, endemic to European countries, is the Puumala virus (PUUV), causing more than 10,000 cases of nephropathia epidemica each year. This mild form of HFRS has a case fatality of less than 0.2%. However, mortality associated with the Dobrava virus (DOBV) in central Europe, and HTNV or Seoul virus (SEOV) in Asia may reach 15%. It is worth mentioning that SEOV has recently been described in wild and pet rats in several European countries [7,8,9,10] and that a few human cases have also been detected in the UK and France [11,12]. The easy adaptation of rats to other territories, in particular urban areas, has led to the extension of SEOV outside of Asia. In the Americas, HCPS presents with pulmonary edema, and although cases are less frequent (hundreds per year), hantaviruses, such as the Andes virus (ANDV) in South America and the Sin Nombre virus (SNV) in the US, can give rise to mortality rates of up to 50% of infected individuals. Despite these variable clinical pictures [13,14], which depend on the hantavirus and its geographic origin, HFRS and HCPS share common characteristics. In particular, thrombocytopenia [15,16] and vascular leakage [17,18] correlate with disease severity. These features have been compared to the different forms of dengue presenting similar clinical manifestations [19].

Many questions concerning the mechanisms of transmission and pathogenesis in humans, and the way hantaviruses propagate in individuals (entry, tropism and targeted organs for diseases) remain to be answered. Understanding how hantaviruses interact with specific host factors may explain: (a) the diverse degrees of pathogenicity observed in humans, from nonpathogenic to nephropathia epidemica, HFRS or HCPS; and (b) how a persistent and asymptomatic state is achieved in natural hosts. Recent specific reviews have addressed hantavirus pathogenesis [14,20], hantavirus interactions with the immune system [21,22,23], or their persistence in rodents [24,25]. These topics will not be detailed here. Rather, the present review summarizes information on pathogenic factors, as deduced from a comparison of the effects of pathogenic versus nonpathogenic hantaviruses in humans and natural hosts. A better understanding of the mechanisms leading to the different hantavirus outcomes should throw light on the physiopathology of these viruses and help to identify factors of pathogenesis that could represent potential therapeutic targets.

## 2. Outcomes of Hantaviruses: Persistence in Rodents and Pathogenicity in Human Hosts

Although hantaviruses are asymptomatic in their rodent reservoir, they are highly viremic and seroconversion takes place in this natural host, as indicated by antibody production, including neutralizing antibodies. Recent data using laboratory animals show that viremic phases in wild rodents are longer than previously described [26]. The virus is found in many organs, such as the kidneys, liver, spleen, heart, and in greater amounts, the lungs. However, the type of infected cells in each tissue or organ has not always been determined. The virus is also found in the saliva, urine and feces of rodents [27], and can persist for several weeks in the environment [28], which increases the risk of transmission to humans. Variations in the levels of virus shedding are observed depending on the hantavirus species. Hantaviruses replicate in natural hosts despite an adaptive immune response. This raises the question of the way tolerance and resistance are balanced in the rodent host such that hantaviruses escape the innate and adaptive immunity and establish a persistent state without being eliminated.

In contrast to what is observed in rodents, human hantavirus infection may lead to clinical signs. Similar to a few other human pathogens causing hemorrhagic fevers, endothelial cells are the main targets of hantavirus infection [29]. In HFRS and HCPS patients, endothelial cells are ubiquitously infected throughout the body, although injury is most prominent in the lungs and kidneys. The infection is non-lytic, but the altered function of the infected endothelium accounts for increased vascular permeability, hemorrhage, acute thrombocytopenia and pulmonary edemas or kidney failure as described for hantavirus diseases [30].

The narrow host specificity of hantaviruses, with one virus species adapted to one rodent species, and the fact that natural rodent hosts do not harbor any obvious symptoms, make it difficult to develop animal models to study the physiopathology of these viruses. The few studies that have been conducted using rodent models of hantavirus disease were performed with the Turkish hamster [31] or the Syrian hamster, in which only pathogenic New World hantaviruses induce symptoms resembling HCPS [32,33]. However, a recent report has shown that the Imjin virus (MJNV) hosted by some Asian shrews was able to induce pulmonary syndromes in Syrian hamsters [34]. The best animal model remains the macaque nonhuman primate for both New and Old World hantaviruses [35,36,37]. Recent data described possible infection of humanized SCID mice with hantaviruses [38]. Today, most data on the physiopathology of hantaviruses come from analyses of human clinical samples, or host reservoir samples, as well as from in vitro studies with cellular models.

The mechanisms underlying the major symptoms of vascular permeability observed in both HFRS and HCPS are not fully understood. In particular, endothelial cells are not directly lysed by hantavirus infection. The current hypothesis to explain the vascular leakage is that an excessive innate immune response could impair the barrier functions of the endothelium [14,39]. It was then proposed that infected endothelial cells from lung or kidney would secrete factors leading to the recruitment of innate immune cells (macrophages, dendritic cells, neutrophils, T cells). These recruited immune cells, in turn, would secrete pro-inflammatory cytokines upon activation, affecting barrier integrity. In addition, macrophages can be infected by hantaviruses, which would also deliver inflammatory factors. In addition, many studies on HCPS have highlighted the role of vascular endothelial growth factor on the regulation of vascular permeability, as is the case following hypoxia [30].

While pathogenic hantaviruses do not always give rise to severe diseases, some serologic evidence has been obtained for human infection by hantaviruses that are considered to be nonpathogenic, like Prospect Hill (PHV) or Tula (TULV) viruses [40,41]. Additional epidemiological studies are needed to clarify whether or not other nonpathogenic rodent- or insectivore-borne hantaviruses may replicate in humans. Understanding how these different viruses interact with human and small mammals such that the endothelium can be infected with or without harboring dysfunction is of great importance. It would be particularly interesting to investigate if the same mechanism operates in asymptomatic rodents and in humans infected with nonpathogenic hantaviruses.

## 3. Hantavirus Propagation in Different Cell Types

Until now, there has been no observed human–human transmission of hantaviruses [6], except in some HCPS cases caused by ADNV [42]. Although bites between fighting males may be a mode of transmission between rodents, the major portal for hantavirus entry into rodents and humans is inhalation of aerosolized viruses. For subsequent maintenance and transmission between individuals, the virus must replicate in target cells, then disseminate from the lung to other organs, and then must be shed.

It is of interest to evaluate whether or not differences can be found in the way each hantavirus species infects its main target cell. Viral envelope glycoproteins, Gn and Gc, play an important role in this targeting process by interacting with specific entry receptors, which can vary according to the hantavirus. Virus particle budding and interaction with cellular factors may also occur in different intracellular spaces during the virus cycle [43].

### 3.1. Entry Receptors

As described for some other viruses, evidence strongly suggests that the cellular entry of hantaviruses is mediated by interaction of the viral glycoproteins Gn-Gc with integrins. These cellular membrane proteins promote cell–cell and cell–extracellular matrix adhesion. They also induce signaling cascades regulating cell proliferation, survival, differentiation, activation and migration [44]. Interestingly, pathogenic and nonpathogenic hantaviruses use different integrin receptors at the surface of human cells: α11β3 is used by hantaviruses inducing HCPS, αvβ3 by hantaviruses inducing HFRS, and α5β1 by nonpathogenic hantaviruses [45,46]. Different cell types express different integrins and the β3 chain is an abundant surface receptor of endothelial cells, dendritic cells (DC) and platelets, which are known to be susceptible to hantavirus infection [47,48]. In addition, DC and platelets are involved in the pathogenic process of vascular leakage and thrombocytopenia [49].

All pathogenic hantaviruses use β3 integrins to enter human cells. This is rather intriguing given their diversity and specific association, each to a different natural host. However, little information is available about the integrin receptors and co-receptors used by the different hantaviruses in their rodent reservoirs. Indeed, natural host reservoirs of hantaviruses are very diverse and the lack of genetic markers and specific detection tools make it difficult to identify receptors or other partners of interaction in the reservoir hosts. Interestingly, Sangassou virus, an African hantavirus, has been recently described and shown to interact with β1 rather than β3 integrin in its Murinae reservoir [50]. It would be interesting to understand whether or not the specificity of interaction with an integrin receptor, and therefore with the type of target cells, could impact pathogenesis. In this respect, the question of which cells express the α5β1 receptor for nonpathogenic viruses remains to be addressed. It has been recently shown that hantaviruses could stimulate human neutrophils (PMN) and that the β2 integrin receptor that is highly expressed by PMN could act as a new entry receptor [51].

Other membrane proteins of the complement regulatory system, such as the decay-accelerating factor (DAF) for HTNV and PUUV, and gC1qR for HTNV, were identified as co-receptors for hantavirus entry [52,53]. These co-receptors together with integrins could also participate in hantavirus tropism and organ targeting, differentiating pathogenic from nonpathogenic viruses. Other cellular proteins, such as the 70 kDa [54] and 30 kDa protein [55], have also been described for HTNV entry.

Whether or not pathogenic and nonpathogenic viruses for humans use a similar entry receptor in rodents and how this might impact virus propagation has yet to be defined. Viral entry is required, but insufficient to account for pathogenesis since both pathogenic and nonpathogenic hantaviruses are able to infect endothelial cells and macrophages. Therefore, interaction with intracellular factors involved in virus replication and assembly might also play an important role in the outcomes of hantavirus infections.

### 3.2. Maturation and Cell Tropism

Envelope glycoproteins are important for entry, and also in many aspects of virus trafficking, maturation and assembly [43]. Interaction of Gn with Gc for the formation of the spike that interacts with entry receptors, or interaction of the cytosolic tail of Gn (GnCT) with the N nucleocapsid [56] that is required for viral assembly must be finely regulated. Interestingly, Old and New World hantaviruses bud on Golgi membranes or the plasma membrane during virus assembly, respectively. Interestingly, different functional domains have been identified on the GnCT of different hantaviruses. A conserved zinc finger domain favoring interaction has been found in the GnCT of both pathogenic, ANDV, and nonpathogenic, PHV, viruses. In contrast, a signal sequence for degradation by autophagy seems specific to pathogenic hantaviruses [57]. Such structures could play important roles in viral assembly and pathogenesis. Finally, ITAM domains known to be important for triggering intracellular signaling in response to receptor activation are present in the GnCT of pathogenic hantaviruses associated with HCPS. This suggests that GnCT could participate in the deregulation of immune and endothelial functions [58].

In vitro, hantaviruses can infect endothelial cells, epithelial cells, and cells from the immune system, such as macrophages, follicular dendritic cells and lymphocytes via attachment of the viral glycoprotein to surface receptors in a cell- and virus-specific way. For instance, pathogenic, but not nonpathogenic hantaviruses may only infect megakaryocytes that have differentiated [59]. Upon infection, human DC could serve both as vehicles of hantaviruses or allow them to evade the immune system. Moreover, infection induces DC maturation and increases expression of β3 integrin, interferon (IFN)-α and tumor necrosis factor (TNF)-α [60]. Macrophages are the second-most important targets of hantaviruses after endothelial cells in both rodents and humans. Despite a low replication rate and release of infectious virus due to inhibition by IFN-α, monocytes/macrophages from peripheral blood are susceptible to PUUV infection [61,62]. Recently, keratinocytes have also been shown to be permissive to hantavirus infection [63], which is of interest considering that hantaviruses can be transmitted by bites between small mammals.

The difficulties in infecting cell cultures with hantaviruses and obtaining virus progeny have led to only fragmentary information on differential mechanisms of virus-entry, maturation and propagation. Indeed, studies are complicated by the great diversity of rodent and insectivore reservoirs, and at the same time the fine specificity of each hantavirus for one host species. Therefore, development of new primary cellular models from natural hosts is of crucial importance [64] for evaluating selective factors in the different host cells. This will be the only way to understand how rodent tissues are persistently infected, and why some organs are predominantly targeted in a virus‑specific manner during human pathogenesis.

### 3.3. Apoptosis and Cell Survival

It is well admitted that hantaviruses are not lytic for their endothelial target cells since no signs of cytotoxicity were reported in vitro in efficiently infected primary cells including endothelial, epithelial, dendritic and mast cells [65]. However, cell damage in PUUV patients presenting with nephropathies is manifested by increased levels of perforin, granzyme B, and LDH, as well as markers of epithelial cell apoptosis [66]. There are conflicting reports on whether or not hantaviruses induce apoptosis in vitro. There were no effects from PUUV recorded on the viability of Vero E6 and A549 cells [67], whereas TULV was shown to induce apoptosis in VeroE6 cells [68]. The effect could depend on the virus species and/or the cell type because a cytopathic effect of different hantaviruses (HTNV, SEOV, and ANDV) was detected in human HEK293 cells [69]. More recently, it was shown that the N protein of HTNV has an antiapoptotic effect in A549 and Hela cells that is mediated by a down-regulation of p53 [70]. Although it could be suspected that apoptosis will not be induced in infected cells of the rodent reservoir, the mechanisms of hantavirus persistence in these natural hosts are still to be defined.

Interestingly, recent investigations are in favor of a protective role from cells infected by hantaviruses through two mechanisms that could promote pathogenesis. The first mechanism is to induce survival of cells of the innate immunity. As already mentioned, a high level of immune cell activation could be detrimental. For instance, a subset of natural killer (NK) cells expressing the NKG2C receptor, specific for the human leukocyte antigen (HLA)-E ligand expressed by endothelial cells, expands in patients during acute PUUV infection. Interleukin (IL)-15 and the anti-apoptotic Bcl-2 molecules both play a role in promoting the survival of these proliferating NK cells [71,72]. Similarly, neutrophils that are now thought to play an important role in vascular leakage observed in hantavirus disease [39] are activated in vitro and in vivo and survived longer in response to hantavirus exposure [51,73]. We also have recently observed prolonged survival of neutrophils due to a delayed apoptosis, specifically induced by the pathogenic PUUV, but interestingly, not by the nonpathogenic TULV or PHV hantaviruses (Baychelier, personal communication). The second mechanism is to protect infected cells from the cytotoxic effect of immune cells. For instance, ANDV and HTNV infected endothelial cells are resistant to NK cell-mediated killing. Consequently, infected cells will be protected, but non-infected surrounding cells will not be protected and are killed by NK cells [74].

Taken together, the picture is still unclear about the effect of hantaviruses on cell survival and how different mechanisms could operate depending on whether hantaviruses are pathogenic or not.

## 4. Interaction of Hantaviruses with the Immune System

The IFN cytokine family is the first line of antiviral defense. Invading viruses are detected by nonimmune cells early during infection. Recognition of viral molecules by pattern recognition receptors (PRRs), such as Toll-like receptors (TLR) or the RIG-I and MDA5 cytoplasmic helicase receptors, modulate signaling pathways, or transcription factors, resulting in the induction of IFN-α/β [75]. IFNs then induce expression of a large pattern of different IFN-stimulated genes (ISGs) to set up an antiviral state. In addition, PRRs directly trigger pro-inflammatory responses that induce host resistance to infection and activate innate immune cells before the establishment of an adaptive immune response. Among the ISGs targeted by viral infection, ISG15, a ubiquitin homolog, MxA monomers which bind and degrade viral components, RNase L which cleaves cellular and viral RNAs, and PKR which inhibits the phosphorylation of the eIF2α translation initiation factor, have been described as directly promoting antiviral states [75].

### 4.1. Induction of the Different Interferon Responses 

Upstream of type I IFN induction, TLR4 expression has been shown to be higher in HTNV-infected human endothelial vein cells (EVC-304) as compared to their uninfected counterparts, leading to an increase of IFN-β, IL-6, and TNF-α secretion [76]. Increased IFN-β production may confer an antiviral state, whereas IL-6 is likely responsible for a pro-inflammatory response. Furthermore, TNF-α, which contributes to endothelial permeability, plays a key role in the pathogenesis of HFRS. Indeed, the permeability of endothelial cells is significantly prolonged upon TNF-α treatment in HTNV-infected cells as compared to uninfected cells [77]. In this same cellular model, the adaptor protein TRIF is up-regulated downstream of TLR4 [78]. Another study, using a human A549 epithelial lung cell line and HuH7 hepatoma cell line, has demonstrated that pathogenic HTNV and nonpathogenic PHV activate innate immune responses in different ways. Both viruses induce IFN signaling via an activation of Mxa, and, in the case of HTNV, but not PHV, this follows the recruitment of TLR3 [79]. These findings are in line with the fact that in endothelial cells, the pathogenic ANDV virus regulates the early interferon response, whereas the nonpathogenic PHV does not. This may explain how PHV can infect EC without being able to replicate [30]. In support of this idea, PHV, but not the pathogenic HTNV, New York 1 virus (NY-1) and ANDV hantaviruses, induces a high level of IFN in human EC early after infection [80]. However, the situation is more complex because another nonpathogenic hantavirus (TULV) replicates as successfully as pathogenic ones in human ECs suggesting that TULV is also able to regulate cellular IFN responses [81].

Type II IFN-γ also exhibits antiviral activities. It has been shown for instance that pre-treatment of Vero E6 and A549 cells with IFN-γ inhibits HTNV infection in a dose dependent way and by a mechanism that is MxA-independent [82]. However, an already-established HTNV infection is insensitive to subsequent addition of IFN-γ stimulation. The same observation has been made for IFN-α/β and IFN-λ [83]. These observations are consistent with the early antiviral effect of IFN on viral replication.

Type III IFNs, which include IFN-λ1/IL-29, IFN-λ2/IL-28A and IFN-λ3/IL-28B, are regulated in a similar manner to type I IFNs [84]. The antiviral effect of IFN-λ has been tested on infected cells. Interestingly, a synergistic inhibitory effect of IFN-λ with IFN-γ, but not with IFN-α/β, was seen on HTNV replication in A549 cells [83]. In line with this finding, a high level of IFN-λ1 is induced in HTNV infected A549 cells, and MRC-5, a human fibroblast cell line lacking the IFN-λ receptor. Expression of IFN-λ1 preceded the induction of MxA and IFN-β. Furthermore, induction of IFN-λ1 and MxA has been observed in Vero-E6 cells [85], which are unable to produce type I IFNs and are used to prepare viral stocks. Three New World hantaviruses (SNV, ANDV and PHV) also induce IFN-λ in Vero E6 cells. Presence of IFN-λ in virus stock prepared from the supernatant of infected Vero E6 cells, has been demonstrated to activate ISG56 and MxA genes in Huh7 and A549 cells. This happens independently of the virus used to infect these cells, as demonstrated by neutralization of the effect using specific IFN-λ antibodies. The situation is different in human umbilical vein endothelial cells (HUVEC) infected by SNV where IFN-λ induction of ISGs is virus-specific, and therefore, not affected by IFN-λ neutralizing antibodies [86]. This is in good agreement with the fact that HUVEC cells, which lack the IL-28Rα chain, are unable to respond to IFN-λ [87]. However, IFN‑λ levels decrease in the serum of PUUV-infected patients, whereas IFN-α/β levels remain unchanged and IFN-γ is elevated. This apparent contradiction could be due to an efficient counteraction of IFN‑λ by hantaviral proteins [83].

### 4.2. How Do Hantaviruses Evade the IFN Antiviral Response? 

In order to successfully replicate in cells, viruses have evolved many mechanisms to counteract the IFN-induced host defense at almost every step of the signaling pathway [88]. This is supported by the fact that pre-treatment of cells with type I IFN-α/β can block hantavirus replication [82,89].

Transcriptional activation of the IFN-β gene requires assembly of an enhanceosome containing ATF-2/c-Jun, IRF-3/IRF-7, and NF-κB [90]. The N nucleocapsid of HTNV blocks TNF-α induced activation of NF-κB by impairing its nuclear translocation [91]. This effect is shared by the N protein of DOBV and SEOV, but not of PUUV, SNV and ANDV [92]. More recently the N nucleocapsid of ANDV has been shown to carry a virulence domain able to inactivate PKR, and therefore counteract its antiviral effect [93].

The S and M segments of some bunyaviruses encode nonstructural proteins (NSs and NSm), which can block IFN-β transcription as shown for the NSs of the Rift Valley Fever virus [94]. Since hantaviruses do not possess NSs or a NSm proteins, these activities must be carried by one of their four structural proteins (N, Gn, Gc or the L polymerase). It should be noted that a few hantaviruses, hosted by rodents of the Arvicolinae family, contain an evolutionary conserved short open reading frame. This sequence could encode a NSs protein that has been shown to influence the interferon response in PUUV-infected cells. It seems unlikely that this NSs represents a virulence factor for human infection since it is found both in pathogenic, PUUV and nonpathogenic, TULV hantaviruses and is absent from highly pathogenic hantaviruses such as ANDV, NY-1, and HTNV [95,96,97]. 

The Gn envelope glycoprotein contains a 142 amino acid long cytoplasmic tail (GnCT) involved in IFN regulation. An interaction of GnCT from the pathogenic NY-1, but not from PHV, with interferon elements inhibits the antiviral action of IFN in endothelial cells [98]. GnCT recruits TRAF3 leading to its dissociation from TBK1. The ubiquitin ligase activity of the complex TRAF3-TBK1 that phosphorylates IRF3 is then abolished along with signal transmission required for the transcription of IFN-β [30,99]. This allows pathogenic NY-1 hantavirus, but not nonpathogenic PHV, to bypass innate immune responses and to successfully replicate within endothelial cells. In contrast to PHV, the GnCT from the nonpathogenic TULV behaves similarly to the pathogenic NY-1 by inhibiting IFN- and ISRE-directed responses upstream of IRF3 at the level of the TBK1 complex. However, unlike pathogenic hantaviruses, TULV GnCT failed to bind TRAF3 [100]. GnCT could be a virulence factor responsible for the delayed IFN responses observed with NY-1, ANDV and HTNV in infected cells. In line with the pivotal role of IRF3 activation in eliciting IFN responses, the nuclear translocation of IRF-3 is impaired upon SNV, HTNV and SEOV infection, whereas nuclear accumulation of IRF-3 is seen with nonpathogenic PHV, TULV and the Thottapalayam virus [101]. Strikingly, the ability of hantaviruses to provoke an early IFN response appears necessary for pathogenesis, but also leads to distinct responses for the two nonpathogenic hantaviruses, PHV and TULV. The fact that the nonpathogenic TULV replicates successfully in EC, suggests the existence of additional viral determinants of pathogenesis [81].

Another way for hantaviruses to interfere with the early innate response, as shown in HTNV infected A549 cells [83], could be at the level of STAT1 transcription which controls the three types of IFNs signaling. Unexpectedly, the glycoproteins of both pathogenic ANDV and nonpathogenic PHV could inhibit STAT-1 nuclear translocation thus impairing IFN signaling. The difference in pathogenicity of these viruses could then be based on different strengths of IRF-3 activation [102] in ANDV and PHV infected human lung microvascular endothelial cells (HMVEC-Ls).

The various outcomes of hantavirus infection could then relate to differential interaction with early steps of the IFN antiviral pathway. Pathogenesis could result, at least in part, from the delayed transcription of IFN-β and other ISGs, allowing pathogenic hantaviruses to rapidly replicate and spread through endothelial cell barriers.

Concerning the role of domains from the GnCT and N viral proteins as a virulence factor, and the fact that hantaviruses are asymptomatic in rodents, one speculation is that interactions of viral proteins with host factors have differently evolved according to the genetic background of their rodent reservoir. This could explain why glycoproteins of hantaviruses behave differently in terms of interaction with elements of the innate immune system leading to different degrees of pathogenesis in humans and to persistence only in specific natural hosts. Altogether, these observations point to the complexity of the mechanisms by which hantaviral proteins interfere with the innate immune system of different hosts, and suggest the involvement of other processes, such as adaptive immunity.

### 4.3. Differences in B and T Cell Immunity Induced by Hantaviruses

Hantaviruses are viremic in humans and in rodents, and neutralizing antibodies are produced in both cases. Antibodies protect humans from re-infection, but obviously do not impair the virus circulation and persistence in rodents. This phenomenon cannot be easily explained and the differential role of T cells has been put forward.

NK cells are at the frontier between innate and adaptive immunity. Under pathogenic conditions, they rapidly expand and then persist during acute infection in humans, with their number remaining elevated for at least 60 days. A strong T cell response involving CD8+ cytotoxic lymphocytes, and to a lesser extent CD4+ T cells, also accompanies the acute phase of PUUV infection [103]. A mixed pattern of T cells of the Th1 and Th2 phenotypes as well as high levels of pro-inflammatory cytokines, not efficiently suppressed by regulatory cytokines, lead to harmful effects in infected patients [22]. Thereafter, T cells decrease with the decline in viral load and the clearance of viremia that are probably due to the effect of intrinsic negative signals and extrinsic regulation.

It is hypothesized that CD4+ regulatory T cells (Treg) can account for viral persistence in rodents [61], whereas NK and CTL have a role to play in human pathogenesis. Presently, induction of regulatory FoxP3+ CD8+ or CD4+ T cells has not been detected in patients during acute hantavirus infection. However, CD8+ and CD4+ T cells may modulate effector responses through different inhibitory receptors during an acute viral infection [104]. It is thought that hantavirus infection induces DC differentiation, and subsequently antigen presenting cell (APC) transition and T cell stimulation. In particular, induction of memory T cells with long lasting protection is found in infected patients [105]. Different infection outcomes in humans and rodents could be explained by the fact that T-CD8+ attracted by infected endothelium would be damaging in humans, while in rodents, these T cells will be inactivated by the induction of Tregs. However, this picture is an over simplification because increased severity of PUUV-induced nephropathia epidemica correlates with higher levels of FoxP3+ Treg [106].

The importance of T cell responses is also attested to by the fact that the major response to hantaviruses is against immunogenic CD8+ T cell epitopes restricted to HLA-I [104]. In this respect, hantavirus infection in different cell types up-regulates the expression of HLA-I, which is involved in antigen presentation, as well as DC cross-presentation to CD8+ T cells [107].

As already mentioned, little is known about the way hantaviruses can replicate and persist in small mammal reservoir species despite the induction of seroconversion and high titers of neutralizing antibodies in these natural hosts. Of note, epitopes on the N and Gn structural proteins recognized by antibodies induced in rodents are different from those inducing human antibody reactivity. In contrast, three epitopes of the Gc protein are immunogenic in both human and rodents. Each hantavirus species also exhibits a narrow specificity for a specific host reservoir and little is known about the mechanism that limits spill over and adaptation to new mammals. It has been shown that deer mice, the natural hosts of pathogenic SNV, can be infected by ANDV, which is also highly pathogenic to humans and asymptomatic in its host reservoir, the pigmy rice rat. In both cases of virus infection, endothelial cells are infected, but deer mice do not present obvious symptoms. However, ANDV is not maintained in deer mice, whereas SNV persists in its specific host. Interestingly, the deer mice immune reaction to infection is different depending on the hantavirus. Persistence of SNV is linked to a low induction of immune cells. In contrast, heterologous ANDV is cleared after induction of a strong B and T cell immune response [108]. This phenomenon must be a consequence of the long-term evolution and adaptation of hantaviruses with their natural rodent hosts [109].

## 5. Activation of Host Cell Factors by Hantaviruses

### 5.1. Differences Associated with HCPS, HFRS, and Nephropathia Epidemica Pathogenesis in Hantavirus Infected Patients

Although differences have been noted in terms of entry receptors, partners for interaction and modulation of the interferon response, a clear representation of the differences leading to the various degrees of pathogenicity associated with hantaviruses has not yet been obtained. This is not surprising considering the complexity of the signaling cascades that are downstream of the IFN, which can be triggered in different innate immune situations. Moreover, the levels of interferon and their receptors will depend on the cell type as for instance the IFN-λ receptor that is only expressed on epithelial cells. Additionally, IFN signaling is connected to other cellular processes such that during the establishment of hantavirus diseases, pro-inflammatory events also impact coagulation and vascular permeability processes as well as on the recruitment of cells of both innate and adaptive immunity.

Studies using high throughput or multiplex technology reveal further complexity. For instance, in one study using the serum of SNV-infected patients presenting with HCPS, the levels of cytokines specific of the Th1 and Th2 responses vary, revealing increases in IL-6, IFN-γ, sIL2R and TNF-α accompanied by a decrease in IL-10 [110]. In another analysis, 68 different cytokines, including chemokines, angiogenic and growth factors, were tested. The most important changes were an up-regulation of IL‑6, CXCL10, CX3CL11, MIF and MIG, and a down-regulation of CXCL12 and to a lesser extent CCL21, 22, 27 and sCD40L [111]. This up-regulation of cytokine expression, could promote tissue migration of mononuclear cells (T lymphocytes, NK, and DC), with leukocytes playing a role in the repair of lung tissue, as well as in increasing endothelial monolayer permeability. Conversely, the down-regulation of cytokines associated with platelet homeostasis is consistent with the thrombocytopenia observed in patients.

For HFRS patients, the serum levels of IFN-γ, IL-10, CCL2, and IL-12 have been shown to be up‑regulated as compared to healthy controls. Variations then depends on the phase and severity of the disease, with IFN-γ and IL-12 relating to mild forms [112]. An up-regulation of uPAR has also been described in PUUV patients. This receptor binds the β3-integrin and can cause proteinuria by acting on the glomerular endothelium of the kidney [113]. As for HCPS, a significant elevation of IL‑6 and TNF-α plasma levels and also of IL-10 has been detected at the onset of the HFRS acute phase [114]. In addition to factors involved in kidney failure (creatinine, C reactive protein and NO), an up‑regulation of these inflammatory cytokines has been confirmed in a macaque model of PUUV infection [115]. An increase in CXCL10 has also been described in the serum of patients infected by HTNV depending on the disease severity [116]. More recently, factors linked to inflammation or coagulation such as tPA and PAI-1 have also been found in the serum of both HFRS patients and macaques experimentally infected with PUUV [117].

### 5.2. Regulation of Cellular Factors Induced by Hantaviruses in Vitro

There are striking differences in the early induction of ISGs in HUVEC that have been infected with either pathogenic hantaviruses that cause HCPS (NY-1) and HFRS (HTNV) or nonpathogenic PHV hantavirus. Pathogenic viruses suppress the IFN response whereas nonpathogenic PHV activates the response. Induction of IL-6, IL-8, and adhesion molecules involved in the recruitment of leukocytes appears specific to the pathogenic viruses [80]. A similar transcriptional analysis revealed different impacts of the pathogenic SNV and nonpathogenic PHV on vascular endothelial cells [118]. In particular, among other genes, CCL5 and CXCL10 appear to be specifically up-regulated by SNV. Human HUVEC cells infected by HTNV, also show an increase in CCL5 and IL-6 as well as an induction of ICAM-1 and VCAM-1 adhesion molecules [119]. A cytokine and receptor screen has been performed with primary DC infected by ANDV. It revealed the induction of pro-inflammatory cytokines amongst which IL-10 and the matrix metalloprotease, MMP9, are able to indirectly affect the permeability of HUVEC endothelial cells [120]. HUVEC endothelial cells and THP1 macrophages have been used for microarray analysis. The effect of nonpathogenic shrew borne (MJNV and Thottapalaiam virus) and rodent-borne (PHV) hantaviruses was compared to pathogenic HTNV. Currently, no human diseases have been associated with hantaviruses circulating in insectivores. It is worth considering that MJNV and Thottapalaiam virus, thought to be nonpathogenic for humans, might induce pro-inflammatory cytokines as does HTNV, but not PHV [121]. Also as already mentioned, MJNV causes diseases in the Syrian hamster model [34]. This could represent a risk for human emergence of hantaviruses from reservoirs other than rodents and requires further attention.

The differential regulation of the innate immune response by pathogenic and nonpathogenic hantaviruses has been evaluated on other cell models. The fact that the chemokine CCL5 is activated in A549 and HuH7 cells infected by HTNV but not by PHV, suggests the involvement of different downstream signaling cascades [79]. Proteomics has revealed a high degree of CXCL10 activation in HuH7 cells infected with PUUV, as compared to non-infected control cells or cells infected with nonpathogenic TULV or PHV (our unpublished data). CCL5 and CXCL10 were also up-regulated in keratinocytes derived cells that are susceptible to HTNV [63].

### 5.3. Differential miRNA Signatures in Hantavirus Infections 

It has been reported that the expression pattern of single-stranded non coding miRNA gene regulators, differ in response to pathogenic (HTNV) versus nonpathogenic (PHV) hantaviruses [122]. This differential expression occurs in HUVEC endothelial, A549 epithelial or THP1 macrophage human cell lines, in a cell-type-specific manner. Moreover, the expression of several miRNAs involved in the regulation of proteins activated during the innate immune response including Mxa, IP-10 (CXCL10), INF-β or RANTES (CCL5), varies inversely in HTNV and PHV-infected cells. Differentially expressed miRNAs also target immune receptor signaling through RIG-I-like, NOD‑like, Toll-like receptors and inflammatory pathways including JAK-STAT, PI3K-Akt and MAPK signaling pathways. It has also been shown that ANDV infection alters the expression of several EC specific miRNAs involved in vascular integrity, adherence and angiogenesis [123].

### 5.4. Persistence of Hantaviruses in Host Reservoirs 

It is important to understand the mechanisms that explain: (a) the persistence and specificity of one hantavirus for a given host species; (b) whether or not spill-over of hantaviruses can occur between species; and (c) if spill over can result in host switching, i.e., in adaptation to a new host. 

As already mentioned, SNV is persistent in its natural host, the deer mouse, which is also susceptible to an experimental infection by ANDV. Gene expression analyses by quantitative real time PCR arrays have shown that these two viruses interact differently with cell factors of lymph node cells from infected deer mice [124]. The expression profiles are coherent in the way B and T cells induce persistence of SNV or clearance of ANDV [108]. For instance, Th2 and IL-4 signaling factors are upregulated in ANDV infected mice, whereas cytokines corresponding to the Th1 and Treg phenotypes are predominant with SNV. Treg activation has also been reported in Norway rats, a natural host reservoir of SEOV, and could contribute to its persistence. In such a situation, pro‑inflammatory mediators are not activated [125].

Together, data highlight the importance of the innate immune response in the different outcomes of hantavirus infections (pathogenic versus non-pathogenic or persistent). The complexity of the balance between different signaling networks regulating cell homeostasis and the time post infection of the study, might contribute to the variability of results obtained in different cellular models.

## 6. Conclusions

The mechanisms sustaining hantavirus pathogenesis in humans or leading to persistence in the animal reservoir most likely result from an interaction of hantaviruses with the immune system. Many data have accumulated, however, much remains to be understood before a comprehensive synthesis can be established. Arguments converge on the role of inflammatory mediators and immune cells targeting the endothelium during pathogenesis. However, some discrepancies exist and may be due to limitations of cellular models, the way hantaviruses are produced in vitro, or the time post-infection of the observation. Transcriptome or proteome analyses support the existence of differences in the relationships of pathogenic and nonpathogenic hantaviruses with their different hosts. The data obtained so far correlate well with some clinical pathogenic markers. These large‑scale approaches will allow for comparative experiments using different viruses in different cell models. These technologies will be fundamental to the discovery of mechanisms that underlie the different ways these emerging viruses manipulate antiviral responses and perturb cellular function. Additionally, these technologies will also help to identify specific factors that could be targeted to counteract pathogenesis and to design specific therapies.
